# Reproductive and family planning history, knowledge, and needs: A community survey of low-income women in Beijing, China

**DOI:** 10.1186/1472-6874-9-23

**Published:** 2009-08-10

**Authors:** Hong He, Truls Østbye, Anne K Daltveit

**Affiliations:** 1 Institute of health studies, School of Sociology and Population Studies, Renmin University of China, Beijing, PR China; 2 Duke-NUS Graduate Medical School, Department of Community, Singapore; 3 Family Medicine, Duke University, USA; 4 Department of Public Health and Primary Health Care, University of Bergen, Norway

## Abstract

**Background:**

The reproductive health status of China's low-income urban women is believed to be poor. Therefore, understanding their reproductive history and needs and improving services provision is very important. However, few studies have been done to assess reproductive health status, knowledge and needs in this low-income population. The purpose of this study is to broadly assess reproductive and family planning history, knowledge and health needs among low income urban women with an aim to informing health services interventions.

**Methods:**

1642 low-income women age 18–49 from Haidian district, Beijing were selected. All were interviewed via a standardized questionnaire in 2006.

**Results:**

Most women reported at least one pregnancy and delivery (97.7%, 98.3%). Deliveries in hospitals (97.3%) by medical personnel (98.5%) were commonplace, as was receipt of antenatal care (86.0%). Nearly half had at least one abortion, with most (56.0%) performed in district hospitals, by physicians (95.6%), and paid for out-of-pocket (64.4%). Almost all (97.4%) used contraception, typically IUDs or condoms. Reproductive knowledge was limited. Health needs emphasized by the participants included popularizing reproductive health information, being able to discuss their reproductive health concerns, free reproductive health insurance, examination and treatment.

**Conclusion:**

Among poor urban women in Beijing, antenatal care and contraceptive use were common. However, abortions were also common. Knowledge about reproductive health was limited. There is a need for better reproductive health education, free medical care and social support.

## Background

Living conditions for low-income urban populations in China is, in general, less favorable than for other urban dwellers. In the largest cities, Beijing and Shanghai, the physical environment is often poor and there is a lack of stable and safe housing arrangements [1,2]. The low-income population in urban areas is mostly comprised of individuals affected by the recent economic transition. They include laid-off workers from state owned enterprises, registered unemployed urban residents, retired workers without pension, and disabled persons who are usually unemployed [3].

Since 1993, China has gradually implemented a system of income guarantees, which provides relief for citizens whose per capita income is under the minimum income level. Between January and June 2003, more than 7.1 billion Yuan (about 855 million US dollars) was distributed to 21.8 million poor urban Chinese people with less than the minimum income level [4]. Still, their access to health care, especially to hospital care, is limited because of the lack of health insurance and disposable income to pay out-of-pocket medical care costs [4].

In 1994, World Health Organization (WHO) defined reproductive health as a state of physical, mental, and social well-being in all matters relating to the reproductive system at all stages of life, without any diseases or discomfort [4]. Reproductive health is a basic precondition for a good life, and also for the health of future generations. General strengthening of reproductive health and of family planning services has repeatedly been stated as a priority for reducing maternal mortality and improving maternal and child health in China [5].

To meet these goals, departments of family planning have been established in all districts of China. So far, little systematic research has been done to assess reproductive and family planning history, knowledge and needs in the low-income population. This population is likely at higher risk of health problems. In spite of greater needs, they may receive less health services. Therefore, a better understanding of reproductive health service use and needs within this population is crucial for improving services and subsequently for improving health, whether through private or governmental action.

The objective of this study was to better understand the reproductive health history among low-income urban women in the Haidian district of Beijing, China. Specifically, we investigated women's reproductive and family planning history, knowledge, needs, and their suggestions for improving their own reproductive health.

## Methods

### Study setting

Participants for this study were recruited from the Haidian district in Beijing, situated in the northwestern part of the greater urban area. It is 431 square km in area, making it the second-largest district in urban Beijing, and home to 2.24 million inhabitants (2000 Census).

Cross-sectional data were used to assess the reproductive health of a low-income urban population in Haidian. Study participants completed one survey in the summer of 2006. To be eligible for the survey, participants needed to be associated with one of the 29 neighborhood committees or streets in Haidian, and have an average monthly income below the official low-income level (330 Yuan or $42 U.S. per month). A list of all eligible individuals was obtained from the Ministry of Civil Affairs. The study was approved by the Human Research Ethics Committee of China, and informed consent was obtained prior to collecting survey data. Data analyses for this report were exempted from full review by Duke University Medical Center's Institutional Review Board.

### Sampling procedures

A list of all neighborhood committees or streets in this district, with their total number of low-income persons, was generated before the start of the study. Due to differences in the proportion of low-income population in the various neighborhood committees or streets, a probability proportional to size (PPS) sampling method was applied to achieve a representative sample [6]. If an individual refused participation, an alternate subject was selected from the same neighborhood committee or street. Among all eligible participants, as identified in the registry for the survey, 3007 persons were randomly selected to be recruited for the study. In total, 2895 subjects completed the questionnaire, including 1025 men and 1870 women. Because of the nature of our research and the significantly shorter questionnaire administered to men, only the 1642 women of reproductive age (aged 18–49 in our sample) were included in these analyses.

### Field work

Interviewers were trained by the researchers prior to entering the field. Researchers also supervised the field work to ensure that standard procedures were followed. The researchers checked all completed questionnaires daily, and mistakes were corrected immediately.

All interviews were scheduled ahead of time, in the company of the neighborhood committee or street administrator. If a participant was unavailable for any reason at the time of the appointment, the appointment was rescheduled. If the participant was unavailable at the second appointment, another person from the same neighborhood committee or street was randomly selected as a replacement participant.

### Questionnaire

The questionnaire was comprised primarily of closed-ended questions that queried participants' basic demographic characteristics, such as age, education, marital status and job status; general self-reported health status; reproductive health history; reproductive knowledge; and health needs. Never-married women are usually reluctant to answer questions relating to pregnancy, abortion, delivery and contraception. As a result, these items were only asked of married or formerly married women. For contraceptive methods, the respondent was asked to report her current most commonly used method of contraception. Post-menopausal women were asked to report the contraceptive method used previously. No clinical examination was included.

### Present study

The present study is based on survey data from 1642 women aged 18–49, representing a sample fraction of 20% of the total low-income female urban population in the district. The main outcome measures were reproductive health history (data on pregnancies, deliveries and abortions) reproductive health knowledge (a series of 11 questions), and health needs. For health needs, the respondents were asked to give priority to a maximum of three of 14 possible choices.

### Statistical analysis

The analysis program Epidata3.1 was used for data entry, checking, and processing. Consistency and range checks were made and errors were corrected in Epidata before exporting to SPSS 11.5 for analysis. Analyses included descriptive statistics, such as frequency tables and inferential statistical analysis in the form of cross tabulations. Main results are presented in tables stratified by age. All statistical tests (chi-square tests for difference between proportions) were two-sided, and p < 0.05 indicated significant differences.

## Results

### Characteristics

Overall, 96.0% were of Han ethnicity, 82.6% had junior middle school education or higher, 77.9% were married, 82.3% were unemployed, 93.1% depended on subsidy for living, and 43.5% were sick or disabled (Table 1).

**Table 1:** Subject characteristics

	*Count (n = 1642)*	*%*
**Age group (years)**		
18~29	141	8.6
30~39	625	38.1
40~49	876	53.3
**Ethnicity**		
Han	1576	96.0
Minority	66	4.0
**Education**		
Illiterate or semiliterate	100	6.1
Elementary school	186	11.3
Junior middle school	642	39.1
Senior middle school	617	37.6
Junior college and above	95	5.8
**Marital status**		
Unmarried	126	7.7
Married	1279	77.9
Divorced	144	8.8
Widowed	92	5.6
**Job status**		
Unemployment	1351	82.3
Employment	291	17.7
**Income source**		
Subsidy	1529	93.1
Other	113	6.9
**Numbers of family members**		
1	74	4.5
2	342	20.8
3+	1227	74.7
**Convenience**		
Both kitchen and toilet	1074	65.4
Either kitchen or toilet	246	17.7
Neither kitchen nor toilet	233	16.8
**General self-reported health status**		
Healthy	746	45.4
"Sub-healthy"	181	11.0
Sick or disabled	715	43.5

### Reproductive health history- pregnancy, delivery and abortions

For married and formerly married females, we tabulated pregnancies, deliveries and abortions by age. Overall, 97.7% had at least one pregnancy, and 47.4% had two or more pregnancies (Table 2). The vast majority of deliveries (97.3%) took place in hospitals and were attended by medical personnel (98.5%), and 86.0% of the pregnant woman received antenatal care. Younger females had fewer pregnancies and deliveries, and were more likely to deliver in a hospital.

**Table 2:** Reproductive history among ever married by age group

	*18~29 years* *(n = 55)*	*30~39 years* *(n = 609)*	*40~49 years* *(n = 848)*	*Total* *(n = 1512)*
**Numbers of pregnancies**				
0	15.4	2.4	1.4	2.3
1	50.0	50.8	50.0	50.3
2	19.2	30.7	31.7	30.8
3+	15.4	16.2	16.9**	16.6
**Deliveries**				
Numbers				
0	4.4	2.6	0.9	1.7
1	93.3	93.1	91.0	91.9
2	2.2	3.8	8.0	6.1
3	0	0.5	0.1**	0.3
Place				
Public hospital	100	95.7	96.6	96.4
Private hospital	0	1.3	0.6	0.9
At home	0	3.0	2.8	2.8
Delivery Personnel				
Medical personnel	100	98.5	98.5	98.5
Family members	0	1.1	0.9	1.0
Relatives and friends	0	0.4	0.6	0.5
Antenatal care (yes)	88.4	87.4	85.0	86.0
**Abortion**				
Numbers				
0	57.8	52.7	53.5	53.3
1	26.7	32.1	32.6	32.2
2	13.3	12.3	11.3	11.8
3+	2.2	2.9	2.7	2.8
Place				
City hospital	50.0	35.7	33.1	34.6
District hospital	38.9	56.0	56.7	56.0
Family planning service station	11.1	8.3	10.2	9.4
Operation personnel				
Doctor	88.9	97.0	94.8	95.6
Assistant doctor	11.1	1.9	3.8	3.2
Nurse	0	1.1	1.4	1.2
Payment				
Free	22.2	18.5	37.7	29.4
Paid 50%	11.1	3.0	8.5	6.3
Paid full	66.7	78.5	53.8**	64.4

*Note*: Numbers in cells are column percentages. Chi-square tests: **p < 0.01 among age groups.

Nearly half of the females had had at least one abortion, and nearly 14.6% had more than one (Table 2). More than half of the abortions were performed in district hospitals, 95.6% by a physician, and 64.4% had paid out of pocket for the procedure.

### Contraception

Only married and formerly married women were included in the analyses relating to contraception (Table 3). The youngest couples were more likely to use contraception. The most common contraception methods were intrauterine device (IUD) and condoms. Sterilization was more commonly reported in the older age group (p < 0.01).

**Table 3:** Contraceptive use among ever married by age group

	*18~29 years* *(n = 55)*	*30~39 years* *(n = 609)*	*40~49 years* *(n = 848)*	*Total* *(n = 1512)*
**Using contraception**	100.0	96.9	97.7	97.4
**Contraception methods**				
Oral contraceptives	2.0	3.4	5.1	4.3
IUD	30.0	45.4	53.4	49.2
Condom	66.0	42.3	30.1	36.5
"Safe" period	0	2.4	2.6	2.4
Sterilization	2.0	6.0	7.7	6.8
Other	0	0.6	1.0**	0.8

*Note*: Numbers in cells are column percentages. Chi-square test: **p < 0.01 among age groups.

### Reproductive health knowledge

A series of reproductive health knowledge questions were asked (Table 4) about harmful effects on health. The majority of women (64.7%) thought abortions were harmful to health, 15.8% and 27.5% women believed that contraception and sterilization were harmful to health, respectively.

**Table 4:** Reproductive health knowledge

	*Count (n = 1642)*	*%*
**Do you think the following are harmful to health? (numbers and % saying yes)**		
Abortion	1062	64.7
Contraception	259	15.8
Sterilization	452	27.5
**Have you received satisfactory information about the following? (yes)**		
Adolescent health care	701	42.7
Prevention of unwanted pregnancy for adolescent	609	37.1
Prevention of STD and AIDS	1146	69.8
Contraception knowledge for newly married	1046	63.7
Healthy pregnancy and delivery	1186	72.2
Maternal health care	1220	74.3
Birth control and contraception for married couples	1215	74.0
Menopausal health care	539	32.8

*Abbreviations: *STD: sexually transmitted diseases, AIDS: Acquired Immure Deficiency Syndrome.

The proportion who felt they had received satisfactory information about reproductive health issues ranged from 32.8% (information about menopausal health care) to 74.3% (information about Maternal health care).

### Health Needs

The respondents were also asked whether they had any suggestions for improvements to reproductive health service or access to service (Table 5).

**Table 5:** Most important health and social needs reported

*Suggestions*	*Count (n = 1642)*	*%*
Popularize information regarding reproductive health	419	25.5
Discuss their reproductive health concerns	405	24.6
Free reproductive health examination every two years	130	7.9
Free treatment for disease in the reproductive system	62	3.8
Provide free medical insurance for reproductive health	47	2.9
Provide home service for the disabled	200	12.2
Improve healthy service communication skills	41	2.5
Eliminate discrimination against disabled people	31	1.9
Eliminate gender discrimination	51	3.1
Strengthen societal support	83	5.1
Increase subsidies	61	3.7
Provide technical training	27	1.6
Provide low-rate loans	16	1.0
Provide help for job searches	70	4.2

Reproductive health needs identified by participants included: 1) popularizing reproductive health information, 2) being able to discuss their reproductive health concerns, 3) provision of free reproductive health care and health insurance, and 4) provision of home aid or assistance services for the disabled. Almost 7.9% of respondents would like the government to provide a free reproductive health examination every two years. Respondents also expressed a need for societal support, including higher subsidies, greater technical training, and assistance in finding employment.

## Discussion

The low-income population in China has gradually increased from under one million in 1997 to 22.36 millions in 2007 [3]. This population presents reproductive health issues that merit attention from the public health authorities. This study provides an overview of reproductive health history, knowledge and needs as expressed by low-income, urban women in a district of Beijing.

The majority (98.3%) of women had had at least one delivery. 6.4% and 0.3% of the women had two and three or more children, respectively. The average number of deliveries among low-income urban women was 1.05 children per person, which is lower than the number (1.43) reported by urban married women in the 2001 national survey [7]. The proportion of older women who had two or more children was 8.1%, which is significantly higher than among younger women (2.2%). This finding is related to family planning policies of China where, in the early 1980s, the central government advocated one child per family in urban areas, with some flexibility for certain regions and for minorities.

As many as 97.3% reported that the delivery had taken place in a hospital, and 98.5% had their child delivered by medical personnel. These results are similar to those reported in the general population [8]. The proportion of women who reported receiving antenatal care at least once was 86.0% in our study, a finding significantly lower than that found among urban married women nationally (96.4%) [9]. However, it was still higher than what has been reported in rural areas of China (85.6%) [9] or worldwide (over 70%) [10]. Antenatal care is essential to detect and follow up early risk factors for pregnancy-related complications and diseases.

Nearly half of all the women surveyed had had at least two pregnancies and one abortion; nearly 15% reported having had an abortion twice; 2.8% reported three or more abortions. 81.9% of married urban women had at least one abortion, and 13.8% had three or more abortions [11]. Almost ninety-eight percent of subjects reported using some form of contraception; this is higher than that reported by married (83.4%) and "floating" married females (90.6%) [12] [Note: the term "floating" is often used in China for individuals moving from rural hometowns to large cities. Even so, abortion rates are high, suggesting that contraceptive methods used were ineffective. Abortion is legal in China, so it may sometimes be used as a method of birth control by individuals with limited reproductive health knowledge. Health education, especially around contraception, should therefore be enhanced in this population, emphasizing the risk of complications from abortions, and that it should only be used when other contraceptive methods fail.

The prevalence of contraceptive use in this population was 97.4%, higher than the national prevalence reported in 1992 among married women (83.5%) [13]. In our study, 49.2% of couples used an IUD and 36.5% of couples used condoms. Guo Sufang et al found similar IUD use (50.6%) among married women in 2003 [14]. Xiao Wanchun found a proportion as high as 73.8% among rural females [15]. The IUD is an efficient, safe, low-cost, and reversible contraceptive method. The proportion reporting having been sterilized in our study was very low (6.8%), and more commonly reported in the older age group. This low rate may be related to increasing government provision of various safe, efficient, reversible, and low-cost contraceptive methods.

Assessing reproductive health knowledge of low-income women is important as this can reveal the extent to which women are aware of various health problems they may face and whether they might seek help. Although the respondents report some reproductive knowledge, it was quite limited. 35.3% of the women did not think abortion is harmful to health; this is lower than in an earlier national survey of urban females (49.7%) [13]. Low-income women have fewer opportunities for getting necessary information, so easier access to reproductive health education should be a priority.

The most frequently reported needs relating to reproductive health were popularization of reproductive health information, being able to discuss their reproductive health concerns, and free reproductive health insurance, medical examination and treatment. Given these women's limited financial resources, they may be less able to pay for health services. Moreover, the women want stronger societal support in general and the elimination of gender discrimination in particular.

Reproductive health is a key component of overall health. Since many low-income women do not seek medical care because of their limited income, strategies for making the medical system accessible and affordable should be developed [3].

This study is one of the first investigations of reproductive health and family planning history, knowledge and needs of urban low-income women in Beijing, and provides a foundation for developing strategies to improve this vulnerable population's reproductive health status. This study's main limitation is the reliance of self report. The study targeted the low-income population – it would have been of interest (but beyond the resources for the current study), to compare the findings to middle and higher-income women. Some events reported may, especially for older respondents, have taken place long ago.

## Conclusion

Among these urban low-income women, antenatal care and contraceptive use were common. However, abortions were also common and reproductive health knowledge was limited. There is need for better reproductive health education, free medical care and social support among these women.

## Competing interests

The authors declare that they have no competing interests.

## Authors' contributions

HH took part in the collection of data, analyzed and interpreted data, and drafted the manuscript. TØ and AKD critically reviewed the analysis and interpretation of data, and contributed to the revisions of the manuscript. All authors read and approved the final version of the manuscript.

## Pre-publication history

The pre-publication history for this paper can be accessed here:


